# Supplementary material for: Tying Together Multiscale Calculations for Charge Transport in P3HT: Structural Descriptors, Morphology, and Tie-Chains
Source: Polymers (Basel). 2018 Dec 7;10(12):1358. doi: 10.3390/polym10121358 (PMC6401820; doi:10.3390/polym10121358)
Supplement: Supplementary file 1 [file polymers-10-01358-s001.pdf]

Supplementary Materials: Tying together multiscale calculations for charge transport in P3HT: structural descriptors, morphology, and tie-chains

Evan D. Miller [1], Matthew L. Jones [1] and Eric Jankowski [1,*]

1. ZINDO and DFT Comparison

In this section, we compare the ZINDO calculation of electronic properties used in this investigation, to a more rigorous DFT method to determine the accuracy of semi-empirical frontier molecular orbital energy calculations for P3HT. We use three representative P3HT chromophore pairs selected from an equilibrated, ordered test morphology, visualizations of which are depicted in Figure S1. The DFT calculations were performed using the B3LYP functional [1] and the 6311++g** basis set [2].

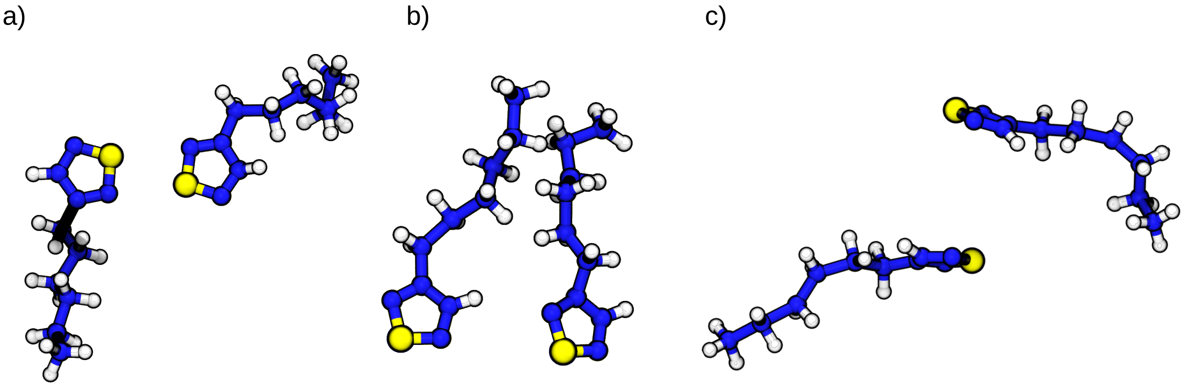

Figure S1. The three representative chromophore pairs used to investigate the accuracy of the ZINDO/S semiempirical method. a) **0469-3714**, b) **0841-1237**, c) **2032-2900**. Terminating hydrogens were added for the QCCs based on position in the thiophene ring.

Table S1. A comparison of the HOMO splitting and calculated transfer integrals for three representative P3HT chromophore pairs

HOMO Splitting	DFT (eV)	ZINDO/S (eV)
0469-3714	0.196	0.095
0841-1237	0.199	0.058
2032-2900	0.086	0.008

The calculated electronic properties of the chromophore pairs are shown in Table S1. ZINDO appears to consistently underpredict the HOMO splitting, which would lead to lower transfer integrals and slower transport than expected from more rigorous DFT methods. However, the ZINDO calculations provide good agreement with the DFT results to within $\sim$ 100 meV, which is already the rough cutoff for DFT accuracy. Furthermore, changes in transfer integral of factors of 2-3 are not expected to significantly affect the charge transport properties given that morphological changes can result in orders of magnitude differences. ZINDO calculations can be performed within 5-10 seconds for a chromophore pair, compared to several minutes to half an hour in the case of more rigorous DFT calculations (depending on the DFT level desired). As such, the computational throughput is significantly improved at the cost of the smaller reduction in accuracy - a critically important point given that each morphology can contain upwards of 40,000 chromophore pairs to be considered.

In summary, the vastly improved computational efficiency at the cost of a small reduction in accuracy of ZINDO/S justifies our use of the semi-empirical calculations for our charge transport properties instead of more rigorous DFT methodologies.

2. Developing ψ' to Explicitly Consider Transfer Integrals

One possible short-coming in our modified order parameter ψ' is that ir is an indirect predictor of the electronic transfer integral $J_{i,j}$ between two chromophores. The hopping rate between two choromophores is strongly dependent on the electronic transfer integral $J_{i,j}$ (see Equation 2 of the main

text) and the energy level difference $\Delta E_{i,j}$ of the two chromophores. Additionally, $\Delta E_{i,j}$ is also partially encoded into $J_{i,j}$, in which chromophores with incompatible energy levels (for instance, a large $\Delta E_{i,j}$) will also reduce $J_{i,j}$ (see Equation 1 of the main text). As such, the transfer integral seems like a good "one-size-fits-all" parameter to describe clustering.

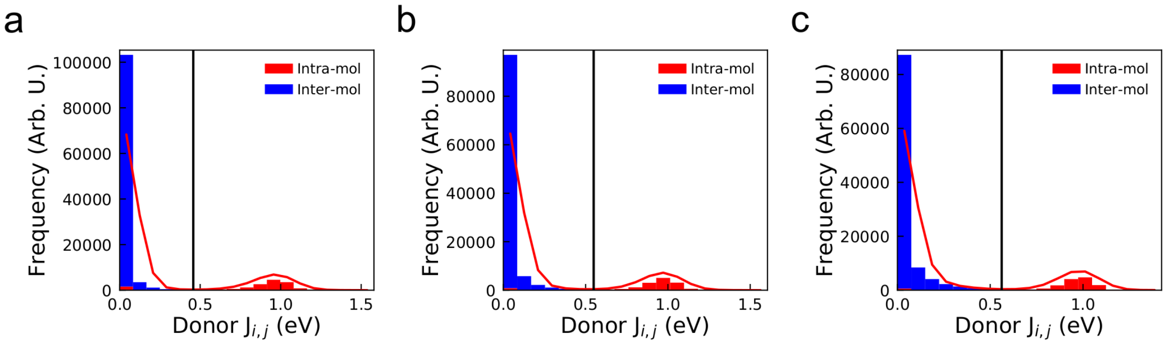

Figure S2. Distributions of chromophore Voronoi neighbor transfer integrals for the representative 1,000 molecule a) amorphous, b) semi-crystalline, and c) crystalline morphologies. The red line shows the Gaussian filtered distribution shape that was used to determine the cluster cut-off criterion. The black vertical line shows the value of the cut-off criterion, which was automatically determined to be at the minimum for each system - $J_{i,j} > 0.562$, 0.549, and 0.457 eV for the crystalline, semi-crystalline, and amorphous morphologies respectively.

The transfer integral distributions for each representative system are shown in Figure S2. In all three cases, the distribution has a large spike at very low transfer integrals and a bump at high TI corresponding to pairs within the same P3HT chain. Initially, we set the transfer integral cut-off to the location of the minimum for each morphology, such that only connections with transfer integrals greater than the cut-off are added to the same cluster. It is convenient to set cut-offs to maxima and minima as these can be determined automatically, rather than being calibrated manually for each separate system. For the crystalline, semi-crystalline, and amorphous morphologies, the cut-offs were set to $J_{i,j} > 0.562$, 0.549, and 0.457 eV respectively.

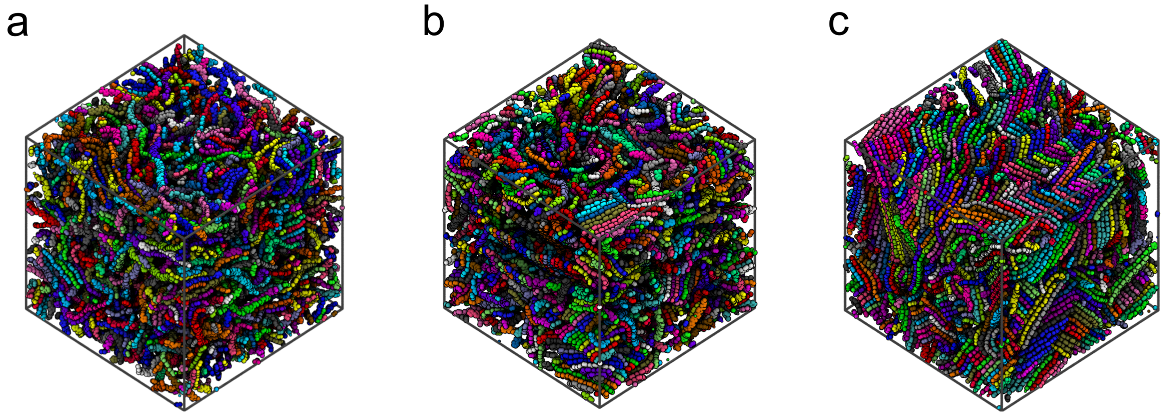

Figure S3. Visualizations of the clusters in the a) amorphous, b) semi-crystalline, and c) crystalline systems with size > 6 monomer units. Clusters were determined based on an automatically-defined transfer integral cut-off for each system based on the distributions in Figure S2.

The resultant cluster visualization in Figure S3 suggests that these cut-off values are too large - in all morphologies, hops with $J_{i,j} >\sim 0.5$ eV are generally only intra-molecular hops (red region in Figure S2). This leads to nearly every chain in the system being considered an individual cluster, with few occurrences of clusters forming between multiple chains. There is no significant difference in the

Table S2. Table of cluster statistics for the three systems, given the automatically-determined transfer integral cut-off criteria.

Property	Amorphous	Semi-Crystalline	Crystalline
Mobility (cm^2/Vs)	1.02×10^{-1}	1.63×10^{-2}	1.16×10^{-1}
$J_{i,j}$ cut-off (eV)	0.457	0.549	0.562
Total clusters (Arb. U.)	1067	1065	972
Large ($>$ 6) clusters (Arb. U.)	964	941	873
Largest cluster size (Arb. U.)	60	60	90

cluster distribution throughout the morphology between the three systems, suggesting that a different transfer integral cut-off should be used.

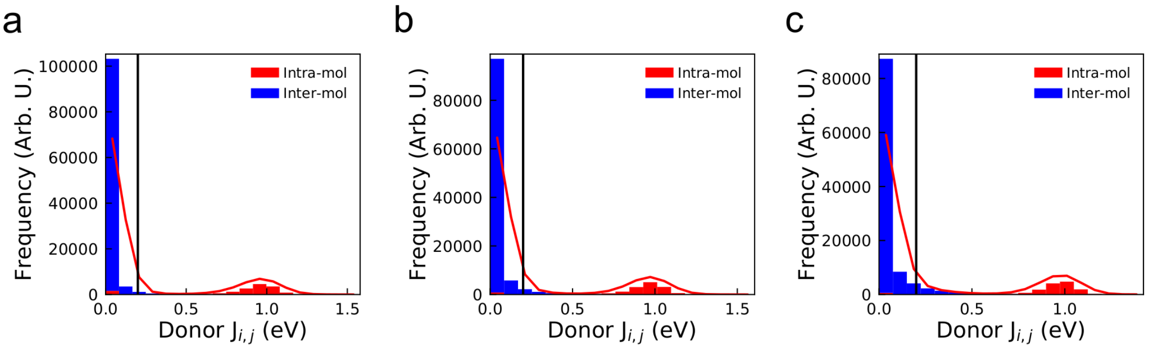

Figure S4. Distributions of chromophore Voronoi neighbor transfer integrals for the representative 1,000 molecule a) amorphous, b) semi-crystalline, and c) crystalline morphologies. The red line shows the Gaussian filtered distribution shape that was used to determine the cluster cut-off criterion. The black vertical line shows the value of the cut-off criterion, $J_{i,j} > 0.2$ eV.

We can, for instance, reduce the cut-off to something smaller in order to include higher $J_{i,j}$ inter-molecular hops. This however, has the short-coming in that such a selection will likely be artitrarily chosen, not rather than an automatically identified minimum. Regardless, reducing the $J_{i,j}$ cut-off to 0.2 eV (Figure S4) provides significantly improved results as now a non-negligible proportion of inter-molecular hops have $J_{i,j} >$ cut-off, thereby, allowing clusters to form between molecules.

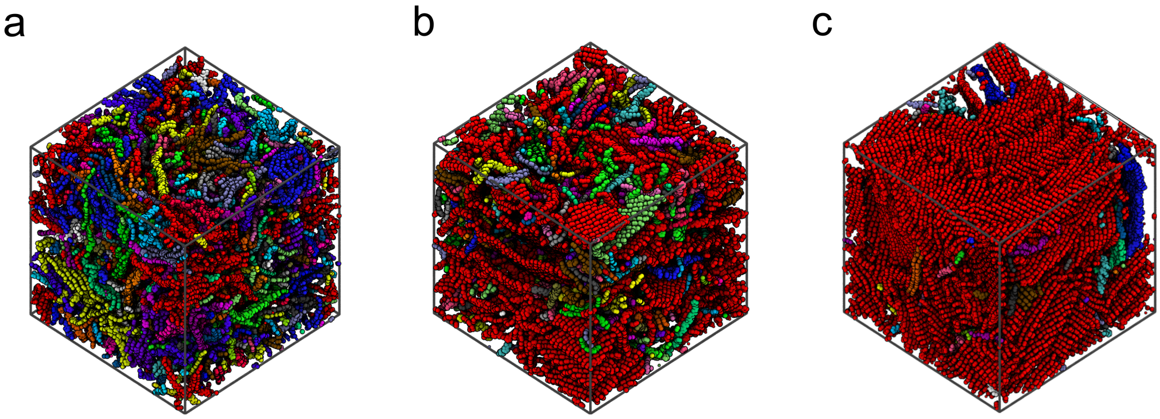

Figure S5. Visualizations of the clusters in the a) amorphous, b) semi-crystalline, and c) crystalline systems with size > 6 monomer units, given the following clustering criteria: transfer integral > 0.2 eV.

Now, we compare the clusters identified with the $J_{i,j}$ cut-off between the three systems. The crystalline morphology shows one large cluster (shown in red) and a few smaller clusters with opposing

Table S3. Table of cluster statistics for the three systems, given the following clustering criterion: transfer integral $J_{i,j} > 0.2$ eV.

Property	Amorphous	Semi-Crystalline	Crystalline
Mobility (cm^2/Vs)	1.02×10^{-1}	1.63×10^{-2}	1.16×10^{-1}
$J_{i,j}$ cut-off (eV)	0.200	0.200	0.200
Total clusters (Arb. U.)	289	163	64
Large (> 6) clusters (Arb. U.)	273	151	51
Largest cluster size (Arb. U.)	2564	9914	12837

grain orientations, indicating that the crystalline system will have a high connectivity. Conversely, the amorphous morphology is predicted to have poor connectivity based on this clustering metric stemming from the larger number of small clusters. Howevever, the connectivity in the semi-crystalline morphology again shows a cluster arrangement intermediate between the other two. This cluster arrangement would again predict a high mobility for the crystalline morphology, a low mobility for the amorphous morphology and an intermediate morphology in the semi-crystalline case, which is contrary to our mobility calculations. Varying the cut-off to any consistent value between the three morphologies always results in this conclusion, suggesting that the transfer integral distribution is not an adequate way to identify clusters in the morphology.

3. Clustering Based on Hops

One short-coming of the previous clustering algorithms is that it considers charge transport between two chromophores in isolation. However, in the KMC algorithm, hops to all neighboring chromophores are considered and the preferential hop (based on the hopping rate between i and j and the random number x) will be chosen. As such, a "good" hop may not occur because there is a better hop.

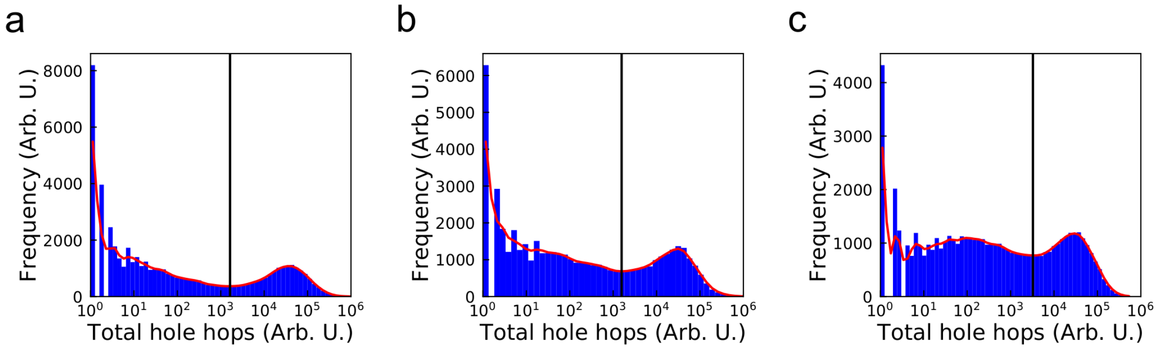

Figure S6. Distributions of the frequencies with which carriers hop between chromophore Voronoi neighbors for the representative 1,000 molecule a) amorphous, b) semi-crystalline, and c) crystalline morphologies. The red line shows the Gaussian filtered distribution shape that was used to determine the cluster cut-off criterion. The black vertical line shows the value of the cut-off criterion, which was automatically determined to be at the final minimum of the frequency distribution: a total of 3264, 1566, and 1635 hops for the crystalline, semi-crystalline, and amorphous systems respectively.

As such, defining clusters based on regions in which charges will freely move is prudent, however, we must still identify a sensible cut-off in hopping frequency to separate these regions. The distributions of total hole hops between chromophore pairs in the three representative systems are shown in Figure S6. Note that the x-axis in these plots is logarithmic, leading to quantization of the bins on the left-hand side of the plot. In all three systems, a second peak appears at high hop frequencies. This leads to a local minimum at 3264 hops in the crystalline case, 1566 hops in the semi-crystalline

case, and 1635 hops in the amorphous case. We therefore use these values as the clustering criteria - only chromophores with connections that are used more than this number during the simulation will be added to the same cluster. We note that the exact values of the cut-off criteria are strongly dependent on the duration of the KMC simulation; the value may change significantly if fewer carriers iterations are performed or if simulation times are reduced. In this study, all three systems used the same simulation time-scales for KMC and the same number of carriers were averaged over in order to obtain the charge transport properties.

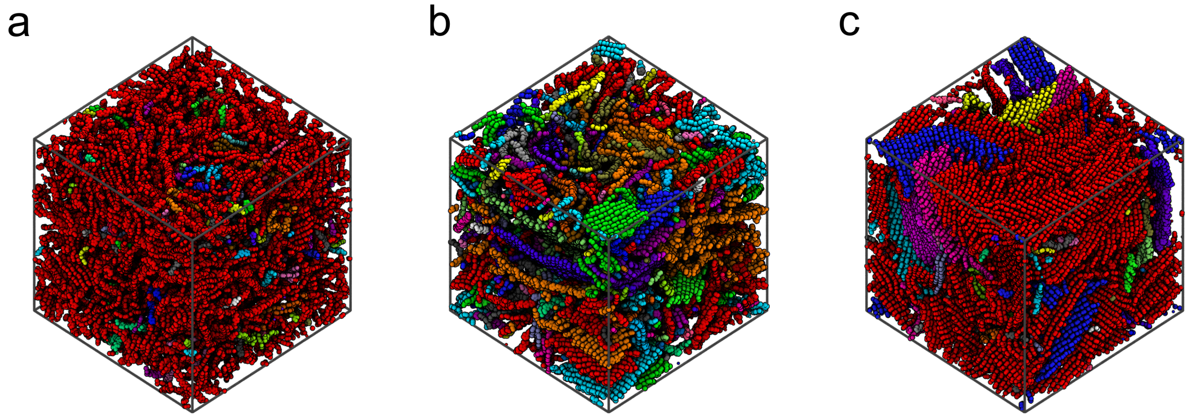

Figure S7. Visualizations of the clusters in the a) amorphous, b) semi-crystalline, and c) crystalline systems with size > 6 monomer units. Clusters were determined based on an automatically-defined hopping frequency cut-off for each system based on the distributions in Figure S6.

Table S4. Table of cluster statistics for the three systems, given the automatically-defined total hop frequency cut-off criteria.

Property	Amorphous	Semi-Crystalline	Crystalline
Mobility (cm^2/Vs)	1.02×10^{-1}	1.63×10^{-2}	1.16×10^{-1}
Hop frequency cut-off (s^{-1})	1635	1566	3264
Total clusters (Arb. U.)	410	1376	418
Large (> 6) clusters (Arb. U.)	134	209	73
Largest cluster size (Arb. U.)	11867	3164	10254

The cluster visualizations using the hop frequency cut-off are shown in Figure S7 are very promising. The crystalline morphology shows different crystalline grains very clearly - although the majority of the simulation is a single cluster (red), a large cluster with a different grain orientation is clearly visible in the morphology (blue). It is harder to distinguish the cluster distributions of the semi-crystalline and amorphous systems using the visualizations, although the crystals present in the semi-crystalline morphology are clearly resolvable from the amorphous matrix surrounding them. However, Table S4 shows the first set of cluster properties where the semi-crystalline morphology is not intermediate between the crystalline and amorphous system, in terms of the number of total clusters and the largest cluster size. These clusters describe regions of the morphology that carriers are frequently hopping within. With this definition, hops within the regions are more common than those between clusters and so carriers are effectively trapped in this region - time is still progressing as they hop around, but their mean squared displacement is not significantly increasing. Therefore, a small number of large clusters is advantageous, whereas a large number of small clusters will strongly restrict charge transport properties.

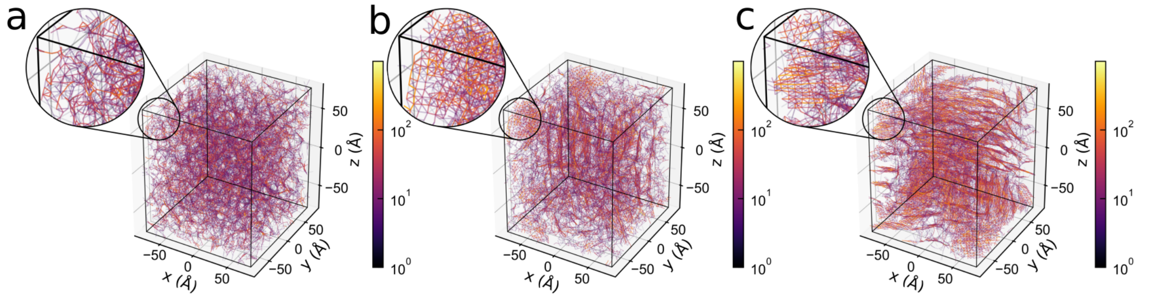

Figure S8. The network diagrams for the (a) amorphous, (b) semi-crystalline, and (c) crystalline systems show carrier pathways between connected chromophores (insets: zoomed regions). Connections are colored based on a perceptually uniform, logarithmic heatmap, where brighter zones correspond to more frequently used pathways.

4. Intra-cluster trapping

In our investigation, we record the location history of every carrier as it hops through the system. Using the carrier hopping history, we can construct network connectivity diagrams (Figure S8) to observe the most frequently travelled paths for charges through the morphology. These network connectivity diagrams are constructed by identifying the frequency with which holes in the KMC simulation hop between pairs of chromophores. The centre-of-mass locations of the chromophores then form the nodes of the network, and the shortest paths between each of the chromophore pairs become the edges. The "net hopping frequency" is calculated by subtracting the frequency of forward hops from backward hops and taking the absolute value. These values are normalized to the highest net hopping frequency in the system, and then assigned a color based on the logarithmic color map to highlight preferred carrier transport routes through the morphology.

The differences in structure between the three classes of morphology are clearly evident in Figure S8. The amorphous network graph (Figure S8a) shows that no crystallites have formed in the system. There are several high-traffic nodes spread homogeneously throughout the system, explaining the highly isotropic carrier trajectory presented in the main text. The crystalline network graph (Figure S8c) shows the lamellar structure of the system, with nearly all chains aligned in layers moving left-to-right across the morphology. The most frequently used pathways are along chains, and there are many connections in the π-stacking direction between chains within the crystal. The semi-crystalline network graph (Figure S8b) exhibits behaviour intermediate between the other two - crystallites with varying grain orientations are clearly visible, within an amorphous matrix.

The insets in Figure S8 show a zoomed region in the corner of the morphology, to highlight an area of 'cross-hatching' in the network, where carriers frequently loop around the same subset of chromophores (located at the vertices of the patterns), without increasing mean squared displacement (MSD) from their initial position. In the amorphous morphology (Figure S8a), no loops are observed, and therefore every hop (no matter how slow) is contributing to the MSD, increasing mobility. In the semi-crystalline (Figure S8b) and crystalline (Figure S8c) systems, significant looping can be seen. Carriers in these regions are becoming 'trapped' by the loops - even though transport may be fast between the chromophores, it becomes more difficult for the carriers to leave the crystal along the slow transport routes due to the wealth of fast hops available within. For the crystalline case, the morphology is dominated by one large crystallite that extends across the full simulation volume. Therefore, carriers getting trapped inside this crystallite are still able to move long distances, and the penalty to the mobility from the trapping is lessened (reflected by a high mobility and a larger anisotropy in the main text). However, in the semi-crystalline case, the morphology is composed of multiple crystallites with various orientations, with loops present across all three dimensions. Trapping therefore has a more significant effect - carriers get stuck in the small loops and are unable to increase their mean squared displacement over time in a single direction, restricting the carrier mobility within the system. These

conclusions are supported by the cluster maps presented in Figure S8, as well as the cluster properties presented in Table 1; the crystalline and amorphous systems are dominated by a single, well-connected cluster of chromophores permitting a high mobility, whereas the semi-crystalline system is composed of many clusters with differing grain orientations. The visualizations of the network in Figure S8 serve to provide additional evidence as to why the clusters described in the main text form within these morphologies.

5. Polydisperse Simulations

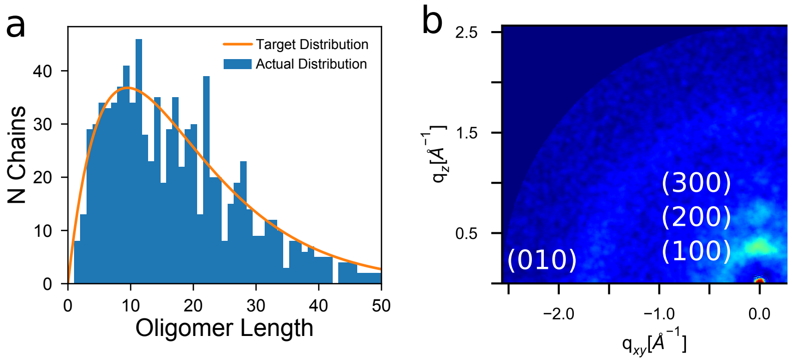

Figure S9. (a) The scaled target distribution of chain lengths and the histogram of the actual chain lengths used in the polydisperse simulations. (b) The equilibrated polydisperse systems is able to produce periodic features not seen in systems where all the chains are longer.

Here we present how we generate polydisperse P3HT simulations. This can be broken into two steps: first creating a dictionary of P3HT oligomers of varying lengths from 1 to 50 monomers long. Second is using a distribution to determine the amount of each chain length to place into the simulation. To produce chains of arbitrary length, we use the open-source program mBuild in which a polymer can be easily created using monomer building blocks. We limit the chain length used in this study at 50 monomers long as to avoid unphysical interactions of chains feeling themselves across periodic boundaries. To generate the distribution of chain-lengths, we use the Schulz-Flory distribution which is a commonly used mathematical description for polymer lengths in the form [3]:

$$P_L = \alpha^2 D_P((1-\alpha)^{D_P - 1}), \quad (1)$$

in which P_L is the probability of seeing a chain of a given length, D_P is the degree of polymerization of a particular chain, and α is a tunable parameter which affects the shape of the distribution. The value for α used in this study was 0.1 and was chosen as this value produces polydispersities of ~ 1.8.

To create the actual distribution of chain lengths we utilize a simple Monte Carlo algorithm. In this algorithm we select a random chain length between 1 and 50 and a random number (x) associated with this chain length between 0 and 1. If x is less than the probability of seeing a chain of that length $P(L)$ we accept the chain otherwise the chain is rejected. In addition to this, to ensure that we have the same number of monomers as the other simulations (15,000 monomers) we keep track of the number of monomers which have been added to the simulation. When the number of monomers added to the simulation via the Monte Carlo algorithm is less than 50 monomers from 15,000 monomers, we terminate the Monte Carlo algorithm and add the remaining monomers to the simulation via one more single chain so that the total number of monomers is 15,000 monomers.

After the Monte Carlo algorithm is finished and a distribution of the chain lengths suggested, we calculate the polydispersity of the simulation with:

$$PDI = \frac{M_w}{M_n}, \tag{2}$$

in which M_w is the weight average molecule weight and M_n is the number average molecule weight. M_w and M_n can be calculated with:

$$M_w = \frac{\sum N_i M_i^2}{\sum N_i M_i} \tag{3}$$

$$M_n = \frac{\sum N_i M_i}{\sum N_i}, \tag{4}$$

in which N_i is the number of chains of that length and M_i is the molecular weight of that chain length. If the PDI of the stochastically generated distribution of chain lengths is below 1.8, we reject the distribution and regenerate the distribution until PDI ≥ 1.8. A comparison between the target distribution and the histogram of chain lengths is shown in Figure S9a.

The distribution of chains presented in Figure S9a is able to produce ordered morphologies with periodic features (Figure S9b) along (100) and (010) signifying π- and alkyl-stacking. These features are seen in experimental and 15mer scattering patterns [4]. However, when simulating systems that contain only 50 membered chains, the system requires much longer to relax into these periodic structures.

Acknowledgments: This work used the Extreme Science and Engineering Discovery Environment (XSEDE), which is supported by National Science Foundation grant number ACI-1053575 [5]. This material is based upon work supported by the National Science Foundation under Grant No. (1229709) and (1653954), and (1658076). The authors thank Chris Muhich for his assistance in comparing ZINDO/S to DFT.

Author Contributions: Funding acquisition, Eric Jankowski; Investigation, Evan Miller and Matthew Jones; Software, Evan Miller and Matthew Jones; Supervision, Eric Jankowski; Writing – original draft, Evan Miller, Matthew Jones and Eric Jankowski; Writing – review & editing, Evan Miller, Matthew Jones and Eric Jankowski.

Conflicts of Interest: The authors declare no conflict of interest.

Abbreviations

15mer - P3HT chain containing 15 monomers
50mer - P3HT chain containing 50 monomers
ε_s - solvent quality
KMC - Kinetic Monte Carlo
MD - Molecular Dynamics
μ_0 - Zero-field Mobility
OPLS - Optimized Performance for Liquid Simulations
OPV - Organic Photovoltaic
P3HT - Poly(3-hexylthiophene)
ψ - order parameter
$\psi\prime$ - modified order parameter
ρ - density
σ - standard deviation
T - Temperature
VRH - Variable Range Hopping
QCC - Quantum Chemical Calculations

1. Becke, A.D. Density-functional thermochemistry. III. The role of exact exchange. *The Journal of Chemical Physics* **1993**, *98*, 5648–5652, [z0024]. doi:10.1063/1.464913.

2. Krishnan, R.; Binkley, J.S.; Seeger, R.; Pople, J.A. Self-consistent molecular orbital methods. XX. A basis set for correlated wave functions. *The Journal of Chemical Physics* **1980**, *72*, 650–654, [arXiv:10.1063/1.438955]. doi:10.1063/1.438955.
3. Flory, P.J. Random Reorganization of Molecular Weight Distribution in Linear Condensation Polymers. *Journal of the American Chemical Society* **1942**, *64*, 2205–2212. doi:10.1021/ja01261a049.
4. Miller, E.; Jones, M.; Henry, M.; Chery, P.; Miller, K.; Jankowski, E. Optimization and Validation of Efficient Models for Predicting Polythiophene Self-Assembly. *Polymers* **2018**, *10*, 1305. doi:10.3390/polym10121305.
5. Towns, J.; Cockerill, T.; Dahan, M.; Foster, I.; Gaither, K.; Grimshaw, A.; Hazlewood, V.; Lathrop, S.; Lifka, D.; Peterson, G.D.; Roskies, R.; Scott, J.R.; Wilkens-Diehr, N. XSEDE: Accelerating Scientific Discovery. *Computing in Science & Engineering* **2014**, *16*, 62–74. doi:10.1109/MCSE.2014.80.
